# Genetic Mutations and Epigenetic Modifications: Driving Cancer and Informing Precision Medicine

**DOI:** 10.1155/2017/9620870

**Published:** 2017-06-08

**Authors:** Krysta Mila Coyle, Jeanette E. Boudreau, Paola Marcato

**Affiliations:** ^1^Department of Pathology, Dalhousie University, Halifax, NS, Canada; ^2^Department of Microbiology & Immunology, Dalhousie University, Halifax, NS, Canada

## Abstract

Cancer treatment is undergoing a significant revolution from “one-size-fits-all” cytotoxic therapies to tailored approaches that precisely target molecular alterations. Precision strategies for drug development and patient stratification, based on the molecular features of tumors, are the next logical step in a long history of approaches to cancer therapy. In this review, we discuss the history of cancer treatment from generic natural extracts and radical surgical procedures to site-specific and combinatorial treatment regimens, which have incrementally improved patient outcomes. We discuss the related contributions of genetics and epigenetics to cancer progression and the response to targeted therapies and identify challenges and opportunities for the success of precision medicine. The identification of patients who will benefit from targeted therapies is more complex than simply identifying patients whose tumors harbour the targeted aberration, and intratumoral heterogeneity makes it difficult to determine if a precision therapy is successful during treatment. This heterogeneity enables tumors to develop resistance to targeted approaches; therefore, the rational combination of therapeutic agents will limit the threat of acquired resistance to therapeutic success. By incorporating the view of malignant transformation modulated by networks of genetic and epigenetic interactions, molecular strategies will enable precision medicine for effective treatment across cancer subtypes.

## 1. Putting Precision Medicine in Context

All cancer treatment is patient-centric and could reasonably be considered to be personalized or precision medicine. For the context of this paper, we have used “precision medicine” to refer to the specific targeting of molecular abnormalities for the stratification of patients to increase responses to specific drugs (Box 1). “Personalized medicine” is within the umbrella of precision medicine; however, these are the most individualized therapies, tailored uniquely for each patient. Precision medicine encompasses drugs that can be used “off the shelf” (e.g., tamoxifen for the treatment of estrogen receptor- (ER-) positive breast tumors), while personalized treatments may require specific engineering for each patient (e.g., chimeric antigen receptor (CAR) T-cells and adoptive transfer of tumor-infiltrating lymphocytes). Both personalized and precision therapies require substantial analysis of the patient's tumor; however, precision therapies, which distribute the burden of development and licensing and testing between patients, are more cost-effective and therefore are likely to be available to a greater proportion of patients [1, 2]. With the expanding availability of high-throughput “omics” technologies and bioinformatic analysis, precision therapy is becoming available to an increasing number of patients. In this paper, we will provide historical context for precision approaches to cancer, review a selection of related genetic and epigenetic contributions to precision medicine strategies, and discuss the challenges and opportunities for the success of precision medicine in cancer therapy.

## 2. Historical Evolution of Precision Medicine

Cancer therapy has historically used an “everything but the kitchen sink” approach. Both Hippocrates and Galen, ancient physicians who have shaped the current practice of medicine, considered cancer as an incurable disease [3]. Cancer treatments have developed substantially since they practiced medicine, which is demonstrated by improvements to patient health and survival.

Treatment for those afflicted with cancers originally used medicines such as extracts from chickpea, adderwort, stinging nettle, and other plants [3]. Surgical approaches to treat cancer have been described as early as the first century A.D., calling for removal of the affected part, accompanied by the now relatively obsolete practice of blood-letting in some individuals [3]. Cancer therapy through the 19th century and most of the 20th century did not deviate substantially from these ancient practices of medicine; however, improved surgical precision, pain management, and sanitation have steadily improved patient outcomes.

Surgical excision to debulk tumors remains a stalwart of cancer therapy, and combinations of medicines, natural extracts, chemicals, and radiation have been introduced in an attempt to limit the rapid cellular growth associated with residual tumor cells. Cancer therapy experienced its first major revolution in the mid-20th century with the use of nitrogen mustards following the observation that mustard gas exposure correlated with depletion of lymphocytes in the blood of soldiers during World War II [4–6]. This prompted a hypothesis that nitrogen mustard compounds could be used to inhibit the growth of white blood cells, which is beneficial to treat and cure leukemias and lymphomas. At the same time, Sidney Farber demonstrated that folic acid could accelerate the growth of leukemia cells. This led to clinical trials of methotrexate, a folate antagonist, to treat leukemia [6, 7]. A third project discovered antitumor effects of the antibiotic actinomycin D, which was used throughout the 1950s and 1960s in pediatric tumors [6, 8]. Finally, a major addition to the therapeutic regime of surgery and chemotherapy that Hippocrates and Galen could likely not have envisioned is the use of ionizing radiation during the 20th century. Observed as early as 1903, Charles Leonard wrote that radiation therapy, initially applied as palliative care, resulted in cures and restored patients' health [9]. Although these approaches improved the survival of patients with cancer, they remained relatively unrefined, with a high risk of acute complications.

Until the late 1900s, cancer chemotherapy used generic cytotoxic drugs that aimed to inhibit rapid cellular proliferation, a characteristic hallmark of malignant cells. The arsenal of cancer chemotherapies expanded to include 5-fluorouracil, vinca alkaloids, platinum agents, and taxanes [10–13], which, though effective at controlling malignant proliferation by inhibiting cellular division, have little precision for specific tumors and often carry high-risk side effect profiles.

**Figure 1:**
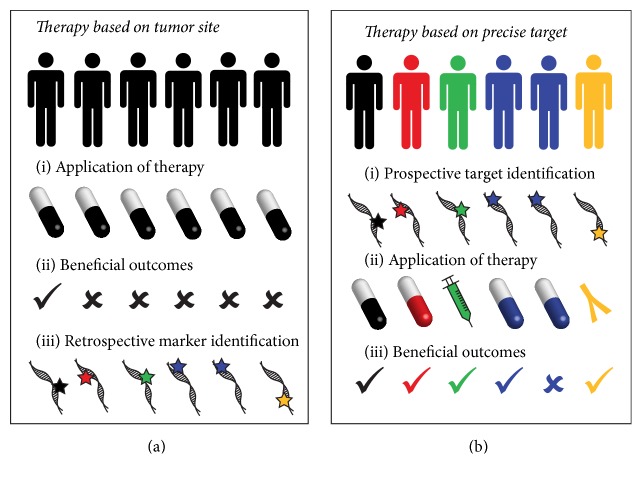
*Retrospective and prospective identification of biomarkers and actionable targets can improve patient outcomes by allowing more precise therapeutic choices*. (a) Traditional treatment of cancers by site of origin. (i) Patients with tumors from the same tissue of origin have typically been treated with the same therapeutic agent. (ii) Treatment outcomes from this type of therapy have been beneficial only in a subset of patients. (iii) With increasing availability of molecular testing, however, we are now retrospectively identifying biomarkers that can predict the outcomes of treatment based on the characteristics of their tumor. (b) Precise patient stratification considers the tumors and the molecular characteristics to determine the best treatment approach. (i) Molecular technologies can identify prospective biomarkers and actionable aberrations. (ii) This allows patients to be given therapies most likely to foster beneficial treatment. (iii) With patient stratification and precise application of therapies, beneficial outcomes are observed in a greater proportion of patients.

Cancer is increasingly considered as a collection of diseases, with characteristics derived from their tissues and cell types of origin, and the mutations that drive them. Chemotherapy, radiotherapy, and surgical therapy are now selected and combined based on their efficacy for particular cancers and histologies, and the process for treating each patient is informed by their specific disease. These strategies to stratify cancer treatment based on the tissue of origin and the specific type of transformed cell were the first refinement toward a more patient-centric treatment of cancer (Figure 1).

The next phase of cancer therapy, precision therapy, will leverage the ability to target specific molecular features to treat a cancer based on its characteristics rather than its tissue of origin alone. More detailed understanding of tumor biology has revealed that each individual tumor accumulates a unique set of alterations that allow it to escape the normal checkpoints that maintain homeostasis. Nevertheless, individual cancers harbour features that allow uncontrolled growth, such as growth factor or receptor overexpression, and loss of apoptosis and cell cycle control mechanisms, which manifest as molecular features that can be targeted with a growing arsenal of drugs. Refining the description of cancer to a unique set of alterations describing each individual tumor will enable precision medicine and inform the best treatment approach to target every tumor and limit the application of ineffective therapies (Figure 1).

**Figure 2:**
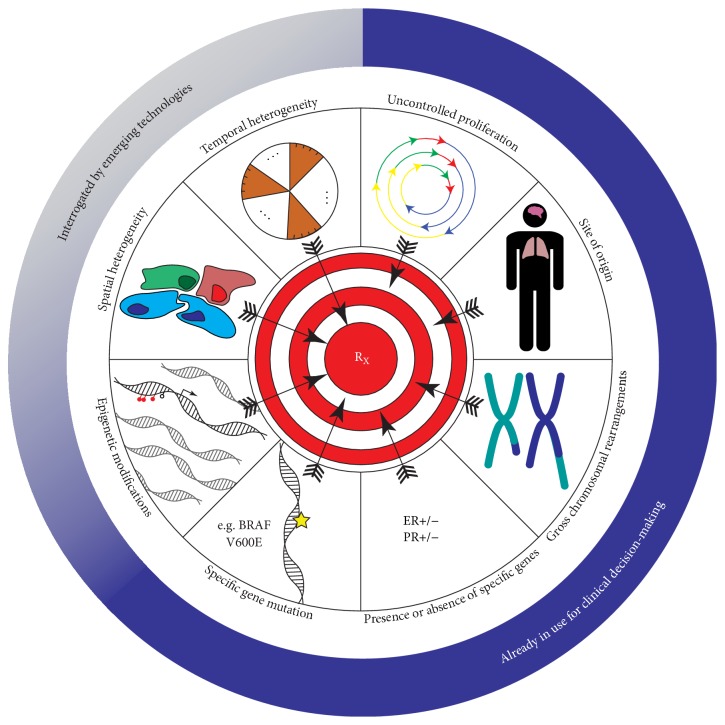
*Emerging molecular technologies provide important information for precision treatment strategies*. Treatment approaches have evolved from a “one-size-fits-all” strategy based on the uncontrolled proliferation of cells and the site of a tumor's origin. Current approaches incorporate gross chromosomal rearrangements and the presence or absence of specific genes which can provide insight into the potential for therapeutic efficacy. Limited precision strategies which target specific mutations are also in use. Emerging technologies will provide a comprehensive view of cancer and allow clinical decision-making and drug development strategies to incorporate epigenetic modifications, spatial heterogeneity, and temporal heterogeneity that can enable acquired resistance to targeted therapy.

Molecular understanding of tissue development, hormones, and required signals for cell proliferation pushed the development of tamoxifen, an estrogen receptor (ER) antagonist, originally developed in an attempt to find new contraceptives and cholesterol-lowering drugs (Table 1) [14, 15]. Tamoxifen was licensed for use in the US for advanced breast cancer patients in 1972, and its current success in treating patients with ER positive breast cancers has been called a catalyst for the precision medicine approach to cancer therapy [16]. The 1990s and early 2000s saw the success of other treatments aimed at molecular targets, such as imatinib against the* BCR-ABL* translocation seen in chronic myelogenous leukemia, rituximab (anti-CD20) as a treatment for B-cell lymphomas, and retinoic acid for treatment of* PML-RAR* fusion acute promyelocytic leukemia (Table 1) [17–21]. In the same decade, trastuzumab was approved for the treatment of breast cancer patients with amplification of* ERBB2* (human epidermal growth factor receptor 2, also known as* HER2/neu*) [22]. More recently, checkpoint inhibiting antibodies, anti-PD1 (nivolumab) and anti-CTLA-4 (ipilimumab) (Table 1), are being systematically applied in clinical trials of particular cancer types [23–25]. While these therapies illustrate the beginnings of cancer treatments that target specific dependencies or weaknesses of cancer, they still largely rely on a site-specific treatment modality (Figure 2).

It is essential to recognize that cancer therapy has historically been limited by our ability to collect and analyze information about the tumor's ontogeny, driver mutations, and phenotype. However, the information we can now obtain from any one patient, and any one tumor, is ushering in a data-driven transformation of oncology which inspires precision therapies. Resources to curate and analyze data collected in whole tumor genomic and transcriptomic analysis, such as the Cancer Genome Atlas (TCGA) and the International Cancer Genome Consortium (ICGC), are making massive cancer datasets available to researchers to test hypotheses and probe genomic variants in primary datasets. Precision medicine is limited by our understanding of cancer and the availability of agents to treat key features of a tumor. An application of “omics” approaches and novel models to understand the complex circuitry of cancer, hijacked from normal cellular networks and pathways, will accelerate improvements in patient care.

## 3. Precision Therapy Is Informed by Genetics and Epigenetics

Two key factors determining cell behaviour are genetics, the study of heritable nucleotide sequences, and epigenetics, which has traditionally been defined as the study of heritable changes to gene expression which do not involve changes of nucleotide sequences. It is important to note that not all epigenetic modifications are heritable [26–28] and thus the term is used more broadly in the context of this review. Canonical epigenetic modifications can alter the transcription and translation of particular genes to increase or decrease their functional levels [29]. Perhaps the most well-studied modification, DNA methylation refers to the addition of methyl groups to CpG dinucleotides in DNA [30, 31] and usually occurs at regions of the genome with a high density of CpG dinucleotides (CpG islands) [32]. Although DNA methylation is canonically associated with gene silencing, the implications of DNA methylation vary significantly with genomic context [33]. Other epigenetic modifications that contribute to transcription and translational control of gene expression include the posttranslational modification of histone proteins (by acetylation, methylation, phosphorylation, ubiquitylation, or sumoylation) [34–36] and the interactions of noncoding RNAs with proteins or other nucleic acids [37]. These epigenetic processes function normally to provide a framework for development and differentiation, contributing to tissue-specific gene expression, inactivation of the X-chromosome, and genomic imprinting [38–40]. Epigenetics also contribute to aging and act in response to environmental factors [41, 42].

As our understanding and treatment of cancer evolve, selection of therapies for cancer patients must also be guided by a molecular understanding of cancer (Figure 2). This includes both genetic and epigenetic factors, as they collude to provide cancer with the required hallmark capabilities [43, 44].

### 3.1. Mutations and Epigenetic Modifications That Drive Cancer

Key alterations, including mutations, are required for the initiation and development of cancer. Broadly, these genetic aberrations enable growth-promoting signals to cancer genes and/or destabilize the genome to allow continuous malignant transformation by facilitating increased rates of mutation [45–48]. It is these mutations that drive cancer and also inform strategies for precision medicine in cancer (Table 1).

#### 3.1.1. Inappropriate Activation of Oncogenes

Oncogenes are most often inappropriately activated by mutations, but the removal of epigenetic marks may also be responsible for activating oncogenes and the rate of mutation varies significantly across cancers of different tissues [49]. The removal of epigenetic marks may also be responsible for activating oncogenes. Hypomethylation of the protooncogenes* HRAS* (Harvey rat sarcoma viral oncogene homolog) and* KRAS* (Kirsten rat sarcoma viral oncogene homolog) was observed in primary human tumors [50]. Although lacking the mutations that would constitutively activate RAS signaling, increased expression of these GTPases may contribute to the malignant phenotype observed in cancers [51] and can be targets of precision medicine approaches (Table 1). Although earlier estimates suggested that 90% of the somatically mutated oncogenes acted in a dominant manner [52], some may instead act in a recessive fashion (i.e., FOXP1 and MLLT4) [53]. The emergence of candidate oncogenes from the comprehensive data available through the TCGA and ICGC databases will likely provide further insight into the relative frequency of somatic mutations in oncogenes and tumor suppressor genes [54, 55].

#### 3.1.2. Genetic Inactivation or Epigenetic Silencing of Tumor Suppressor and Genomic Stability Genes

The majority of tumor suppressor genes require mutations on both alleles [52], resulting in the inactivation of a gene product that controls excessive cell growth under healthy homeostatic conditions. Typically, loss of cellular integrity followed by persistent and uncontrolled cellular division requires inactivation of protective cellular mechanisms (i.e., tumor suppressor genes) and mutations that facilitate tumor growth either directly (i.e., by activation of an oncogene) or indirectly (i.e., by enhancing sensitivity to growth factors). The “two-hit” hypothesis, first described by Knudson in the 1970s [56, 57], postulates that at least two mutations of a tumor suppressor gene are required for cancer to develop: first, loss of heterozygosity, and then subsequent mutation of its paired allele, leading to functional loss of the repression of cell division and cancer development. While the classical definition of a tumor suppressor includes the presence of truncating mutations [58–60], there is evidence that tumor suppressor genes are inactivated by nontypical mechanism such as haploinsufficiency or gain-of-function isoforms [58]. Classical tumor suppressor genes, typically defined by inactivation by mutation, may also be silenced by epigenetic mechanisms [61–64]. We and others have posited that these “silenced” tumor suppressors may make excellent drug targets as they could potentially be reactivated by reversing these silencing modifications [65, 66] or may indicate the pathways and downstream alterations that may be targeted by precise therapeutics.

Genomic instability is a key enabling characteristic underlying carcinogenesis and continuous tumor progression. TP53 plays an essential role in this process as the “guardian of the genome” [67] and is implicated in almost every type of human tumor at varying rates [68]. Broadly, inactivation of TP53 and other gene products responsible for protecting the integrity of cellular DNA support a more mutable phenotype by preventing DNA repair or by allowing mutagenic molecules to damage DNA unchecked [44]. Epigenetic aberrations can also impact the mutation rate of cancer cells. For example, hypomethylation near guanine quadruplexes increases the rate of DNA breakage and activation of homologous recombination and may act as a mutagenic factor [69]. There is also evidence that mutation rate varies near CpG islands and that histone methylation levels may predict the loci of somatic mutations [70–72]. In addition, normal epigenetic regulation can impact the mutation frequency as highly expressed genes have been demonstrated to have higher mutation rates [73].

The effect of epigenetic modifiers on genomic stability is most frequently observed in hematological malignancies. In acute myeloid leukemia (AML) and myelodysplasia, mutations in isocitrate dehydrogenase (IDH) 1 or 2 disrupt cellular differentiation and drive leukemogenesis [74]. Mutations in the catalytic domain of* EZH2* are found in diffuse large B-cell and follicular lymphomas and disrupt H3 K27 methylation, promoting cell survival (Table 1) [75, 76]. Similarly, a common target of translocation in mixed-lineage leukemia is DOT1-like histone H3 K79 methyltransferase* (DOT1L)*. Inhibiting DOT1L results in increased differentiation and apoptosis of leukemia cells, again suggesting that the abnormal epigenetic program is required for leukemic cell survival [77]. Together, this evidence suggests that interactions between the genome and epigenome occur throughout malignant transformation.

Cancer is typically broadly characterized by genome-wide DNA hypomethylation and promoter hypermethylation [78–82]; however, the view and interpretation of epigenetics in the context of precision medicine should be expanded beyond a gene-centric view. The epigenome is not just a surrogate for mutations and can have distal effects which range beyond canonical promoter methylation [72, 83]. Evidence suggesting that methylation of distal regulatory elements is related to gene expression poses a complex question that genome-wide studies are now beginning to answer. The hypomethylation observed in cancer often occurs at satellite DNA, the main component of functional centromeres, and at other repeating sequences that do not function as transcriptional units. Hypomethylation in these DNA sequences is not likely to have a* cis* effect on gene expression, unless it spreads into neighbouring chromatin [78]. However, gene expression can be affected by nuclear positioning, and hypomethylation near centromeres could affect gene expression in* trans*. Centromeric heterochromatin has been shown to act as a reservoir for transcriptional control proteins that may be disrupted by hypomethylation [78, 84, 85]. This hypomethylation may also disrupt interactions between heterochromatin and euchromatin [86–88]. With respect to gene-specific hypermethylation, several studies have observed that most hypermethylated tumor suppressor gene-associated CpG islands are not in gene promoters. This hypermethylation is thus likely to be a consequence of another cancer-associated mechanism rather than a direct cause of tumor development or progression [89–91]. This necessitates that cancer epigenetics step outside of a gene-centric focus and overcome the greater challenges associated with determining the effects of global epigenetic aberrations.

## 4. Precision Approaches to Genetic and Epigenetic Events in Cancer

### 4.1. Targeting Genomic Drivers of Cancer Progression

Our knowledge of driver mutations and oncogene addiction in many types of cancer has prompted development of cancer therapies targeted at molecular changes (Table 1). Although many cancers have multiple genetic abnormalities, driver mutations enable outgrowth of cancerous populations. Changes that foster cancer growth reflect an “oncogene addiction” or reliance of some cancers on one gene, or a few genes, to maintain a malignant phenotype. Addiction to a specific alteration such as overexpression of growth factor receptors may represent an “Achilles heel,” a specific and identifiable weakness which may be exploited by cancer therapies. Indeed, the addiction of folate receptor-positive tumors to folate and the success of the anti-HER2 antibody trastuzumab have been described as “convincing and clinically relevant evidence” for the theory of oncogene addiction [92, 93].

In some tumors with high mutational load, it can be challenging to identify specific mutations as driver or passenger mutations. Driver mutations are those which enable the continued development and evolution of a tumour; passenger mutations result from genomic instability and other tumor-associated factors but are dispensable for tumor progression [94]. For instance, DNA polymerase regularly stutters in short tandem repeats of mono-, di-, tri-, or tetranucleotide repeats [95]. When DNA repair is defective due to silencing of, or point mutations in, key DNA stability genes (such as* MLH1*,* MSH2*,* MSH6*, or* PMS2*), these mistakes cannot be repaired. This results in microsatellite instability and can lead to a hypermutable phenotype [96].

The boom in molecularly targeted drugs has yielded an impressive list of targeted drugs in clinical trials, with many more following behind in the development pipeline. More recently emerging is the off-label use of drugs for the treatment of cancers with specific alterations. For example, integrative genomic analysis of a patient with metastatic colorectal cancer revealed high expression of components of the activating protein-1 (AP1) complex (Table 1). Treatment with the angiotensin II receptor antagonist, irbesartan, resulted in a complete radiological response [97]. This may be an effective therapy against other tumors with a similar upregulation of the AP1 complex, but it is unlikely to be effective for any other alterations. It is important to recognize that the components of* AP1* were not mutated in this patient and were revealed as outliers via gene expression analysis. Thus, the repertoire of molecularly targeted drugs may need to encompass every possible gene product and not just those frequently mutated or rely on approaches to identify mutations a priori in each patient. This concept has driven the development of “basket trials” based on the hypothesis that the individual alterations in a patient's tumor will dictate the response to therapy independent of the histology [98].

One challenge to the concept of oncogene addiction and the premise of molecular targeted therapies, seen even in basket trials, is a maximal response rate of approximately 50–60% [22, 92]. This suggests that the presence of a specific abnormality is not entirely responsible for the phenotype of a cancer and supports an evolutionary model of cancer development where the initial transformation and subsequent tumor growth foster further mutations and epigenetic alterations. Integrative analysis of genetics and epigenetics may hold clues to this missing link.

### 4.2. Epigenetic Therapies and Precision Epigenetic Medicine

The same qualities that make epigenetic heterogeneity difficult to measure and model also make epigenetic modifications excellent drug targets; in particular, the reversibility of epigenetic modifications makes them attractive targets [99]. The concept of oncogene addiction can be mirrored in several descriptions of the aberrant epigenetic landscape found in cancer [100–102]. Cancers may become dependent on the silencing of a few crucial tumor suppressor genes. For example, epigenetic silencing of a negative regulator of the Wnt pathway results in constitutive activation of Wnt signaling, driving proliferation [103, 104]. In another model, epigenetic silencing of* HIC1* (hypermethylated in cancer 1) resulted in a partial loss of p53 function, cooperating to drive tumor growth and progression [105].

Before exploring precision targeting of epigenetic aberrations, it is important to consider the conventional uses of epigenetic therapy. Epigenetic therapy is perhaps most well known for its effectiveness in treating myelodysplastic syndromes (MDS). MDS represents a heterogeneous group of disorders characterized by bone marrow failure. Approximately one-third of MDS patients progress to AML, and this shift is associated with accumulation of epigenetic modifications within the cancerous cells. Many genes have been described as inappropriately silenced in MDS and AML; however, the mechanisms by which hypermethylation of these and other genes contributes to MDS or AML are not particularly well characterized [106–108].

Azacytidine (5-azacytidine) has been shown to be effective in approximately 50–60% of patients with MDS [109, 110]. Azacytidine is a nucleoside analog which is incorporated into RNA and DNA during transcription and DNA replication. When incorporated into DNA, it acts as a DNA methyltransferase (DNMT) inhibitor by binding DNMT leading to an irreversible loss of activity and its degradation [111–113]. Additionally, its structure prevents the addition of methyl groups to DNA [114]. Azacytidine is also known to induce apoptosis, and it is so far unclear if its efficacy in MDS is due to demethylation or due to increased apoptosis [115, 116]. Decitabine (5-aza-2′-deoxycytidine) can only be incorporated into DNA and results in demethylation of DNA and apoptosis in a similar manner to that of azacytidine.

Recent attempts to combine epigenetic therapies with conventional chemotherapies have shown promise. It may be possible to reverse epigenetic modifications that confer resistance to chemotherapy, such as the silencing of* APAF1* in metastatic melanoma. APAF1 is a downstream effector of* TP53* in DNA damage-induced apoptosis and a key mediator of intrinsic apoptosis, and it is frequently downregulated by DNA methylation in malignant melanoma, although it is not known if this is a direct or indirect effect [117, 118]. Some antiapoptotic genes such as BCL2 have been observed to be overexpressed, contributing to chemoresistance. While the source of the aberration is not known, it may be countered by the use of venetoclax, as in chronic lymphocytic leukemia [119]. The impact of reversing the epigenetic alterations which downregulate or upregulate specific genes contributing to chemoresistance (such as BCL2) has yet to be completely characterized; however, combinations of epigenetic agents with chemotherapies have shown clinical benefit [120, 121].

Epigenetic reprogramming in cancer also contributes to the inappropriate expression of tissue-specific genes, such as the cancer/testis antigen group. These are so named because their expression is limited in normal tissues to the testes. These are silenced in healthy cells as a result of DNA methylation [122], but they can be found in numerous cancers [123, 124]. One group of these genes, melanoma-associated antigen genes (MAGEs), are found expressed at high levels in melanoma and squamous cell lung cancers [125]. The expression of cancer/testis antigens is an attractive target for immune-mediated therapy as targeting antigens with limited expression profiles would result in limited off-target effects. As intracellular proteins, they are considered as targets for vaccines rather than antibody-based immunotherapy. Responses to MAGE peptide vaccines, however, have been proven to be fairly limited, and combination therapy with epigenetic modifiers which increase tumor expression of MAGEs may be more effective [126].

The expression of cancer/testis antigens allows specific, engineered T-cells to mediate an antitumor immune response, as has been seen in a clinical trial of personalized medicine for patients with myeloma [127]. Additionally, a recent study has illustrated that demethylation of DNA can upregulate hypermethylated endogenous retrovirus genes and induce the expression of double-stranded RNA, which can mimic a viral response [128, 129]. These phenomena signal that resetting the epigenome via pharmacological inhibition may sensitize cancer cells to other immunotherapies. In a preclinical model, DNMT inhibition sensitized melanoma to treatment with the anti-CTLA4 antibody, an immune checkpoint inhibitor [128], and incidental findings in non-small cell lung cancer support the addition of azacytidine to anti-PD1 checkpoint inhibitor therapy [130, 131]. The complex interactions between the epigenome and the genome suggest that this approach is a promising method to enhance the efficacy of existing precision therapies.

One of the reasons that the epigenome is difficult to precisely target is that it has been challenging to distinguish driver alterations from passenger alterations. While recent evolutionary tracing has contributed to our understanding of driver and passenger mutations in tumorigenesis [132, 133], we are only beginning to identify the cancer-causing epigenetic changes among the many thousands of less-relevant alterations that are a consequence of cancer progression. One notable study identified the gene promoters whose DNA methylation is required for survival of somatic cells, cancer cells, and cells in culture by examining promoter methylation after disruption of DNA methyltransferase activity [134]. The genes identified as necessarily silenced for cancer cell survival were not known classical tumor suppressor genes. This study demonstrated that cancer cells can indeed be “addicted” to specific epigenetic alterations and require silencing for survival [99]. Additionally, hypermethylation of some genes is required only in cell culture [134], providing further insight into a culture-specific phenotype and offering reservations about epigenetic data collected only in culture. Stable immortalized and tumor cell lines display marked hypermethylation of CpG islands, and studies of cultured cells risk revealing artefacts of the culturing process [135].

For the most part, the mechanisms behind the therapeutic benefit of DNMT inhibition and HDAC inhibitors are not fully understood [99]. It has been difficult to model and measure the effects of histone modifications, as the complex, combinatorial nature of the histone code means that each modification must be considered in context with other modifications [35, 136]. Ongoing and future studies which discriminate between driver and passenger alterations offer important insight into the specific alterations which must be targeted to enhance the clinical efficacy of epigenetic therapy. Developing epigenetic inhibitors which target specific genes or groups of genes, such as those genes identified by De Carvalho et al. (e.g., RAK3, P2RY14, CDO1, BCHE, ESX1, and ARMCX1), would overcome the significant risks and side effects of the epigenetic agents currently in use which have global effects [134].

Epigenetic information can also be used to predict clinical outcomes and patient responses to specific therapies [137]. For example, methylation of the DNA repair enzyme O^6^-methylguanine-DNA methyltransferase gene* (MGMT)* in glioma predicts a better response to alkylating agents commonly used in therapy [138]. When expressed, MGMT rapidly reverses the damage caused by alkylating agents (e.g., temozolomide) and confers resistance to therapy (Table 1) [139, 140]. Other epigenetic profiles have been used to prioritize or exclude treatment strategies; however, they lack the clear mechanistic connection seen between MGMT and alkylating agents. For instance, in non-small cell lung cancer, an unmethylated* IGFBP3* promoter is correlated to response to cisplatin-based chemotherapies [141], and methylation of* PITX2* predicts the outcomes of individuals with early-stage breast cancer following adjuvant tamoxifen therapy [142]. These and other markers have not shown a high degree of sensitivity or specificity, perhaps because there is no clear mechanism connecting the gene affected by methylation and the response to therapy. The addition of methyl groups to cytosine is neither necessary nor sufficient to alter gene expression; thus, the measurement of DNA methylation as a surrogate of gene expression or regulation may not be an accurate tool to predict response to therapy [143].

In hematological cancers, where mutations in epigenetic regulators are frequent, targeted therapies show significant promise. In EZH2-mutant lymphoma, selective EZH2 inhibitors have been shown to induce apoptosis, with minimal effects on EZH2 wild-type cells [75]. The use of EZH2 inhibitors decreases the global H3 K27 trimethylation levels and reactivates genes silenced as a result of mutant EZH2 (Table 1) [144]. Preliminary data from the use of an EZH2 inhibitor, tazemetostat, in clinical trials of mutant and wild-type EZH2 lymphoma, demonstrated a favorable safety and efficacy profile to warrant a phase II trial stratified by mutation status [145]. Similar approaches have been seen to target mutant IDH1 in MDS and AML and mutant DOT1L in MLL [77, 146, 147]. While these agents have shown clinical success, their use is challenged by the observation that epigenetic modifiers exist in complexes and may target nonhistone proteins.

True epigenetic therapies have yet to cross the threshold into precision targeting. The majority of histone modification inhibitors are not yet specific enough to precisely target specific alterations [148]. The resurgence of DNA editing and its clinical applications via the CRISPR/Cas9 system opens the door to targeting DNA sequences which are permissive to epigenetic modifications [149–151]. Deposition or removal of DNA methylation marks or of chromatin modifications can have a direct impact on gene expression (reviewed in [152]). It remains to be seen how effective these strategies will be for precision epigenetic targeting in animal or ex vivo models.

## 5. Challenges and Opportunities for the Future of Precision Medicine

The success of precision therapies, regardless of their molecular targets, depends on three key factors: the identification of patients who will benefit from targeted therapies, the ability to determine if a therapy is successful during the course of treatment, and strategies to combat resistance to targeted therapies. The study of exceptional responders, the use of “omics” technologies, and approaches to predict and respond to therapeutic resistance are opportunities to address these significant challenges in the effective implementation of precision medicine.

### 5.1. Patient Identification for Precision Approaches

The identification of patients who will benefit from therapy has been an enormous challenge even for population-based approaches to cancer therapy. Research has sought to moderate the use of aggressive therapies by identifying patients who see no increased benefit from aggressive courses of therapy with tools such as Oncotype DX, a tool for identifying precision approaches to treat breast cancer [153], or SnapShot, a protocol for identifying driver mutations in non-small cell lung cancer [154]. Another example of this is the characterization of patients with medulloblastoma, where molecular characterization has revealed four distinct molecular subtypes: Wnt, sonic hedgehog (Shh), and group 3 and group 4 tumors [155]. Patients with the Wnt subtype exhibit long-term survival of approximately 90%, making them ideal candidates for trials which reduce the intensity of current standard-of-care therapy [156]. This is particularly important as medulloblastoma primarily affects children, and the side effects of chemotherapy and radiation therapy can be quite severe and have lasting impacts. Identifying biomarkers and prognostic patient profiles will allow physicians to choose the most appropriate course of therapy to make the use of existing generic therapies more precise and minimize the morbidity associated with imprecise treatment approaches (Figure 1).

#### 5.1.1. Exceptional Responders

Perhaps the greatest opportunity to identify patients who will benefit from current precision therapies is by study of exceptional responders. It is hypothesized that most clinical trials showing modest benefit demonstrate substantial variability among patients (Figure 1) and could be separated into patients who respond exceptionally well to therapy and patients who fail to respond [157]. A prime example of this is the relative success of trastuzumab in treating HER2-positive breast cancer. Roughly 25–30% of all breast cancers are classified as HER2-positive, and response rates to trastuzumab among this group range from 15 to 80% (reviewed in [158]). Thus, we could reasonably hypothesize that a larger study which did not select patients with HER2 amplifications and instead included all breast cancer patients would have concluded that approximately 4–20% of patients (compared to 15–80% of HER2-positive patients) would benefit from clinical use of trastuzumab. The same can be hypothesized for many clinical trials that fail to show any statistically significant benefit: there are patients who demonstrate an objective clinical response [159, 160]. These patients are deemed exceptional responders, and new initiatives aim to profile these individuals to determine what differentiates these outliers. By definition, exceptional responders are rare, and studies of these outliers in the use of ineffective drugs or those with limited efficacy will lack statistical power [161]. This is a challenge that clinical trials of the most precise therapies and stratification approaches will face. It will be difficult to determine signal from noise even through a comprehensive analysis of sequence data, expression data, epigenetic data, and clinical outcomes [161].

One approach may be to view cancer as “a disease of pathways” instead of examining specific distinct genetic or epigenetic alterations [48, 161, 162]. An integrative, network-based approach which includes all of the factors affecting cellular phenotypes (including DNA and mitochondrial DNA sequencing, DNA methylation, histone modifications, noncoding RNA, gene expression, protein expression, and posttranslational modifications) will not only inform precision treatment but also provide a framework of the hijacked cellular circuitry seen in cancer [163].

### 5.2. Tumor Heterogeneity Is a Challenge for Precision Medicine

Mutations or other aberrations in the expression of genomic stability genes such as those involved in DNA repair or induction of apoptosis can drive hypermutable phenotypes in tumors. Some colorectal cancers have been found to have upwards of 100 mutations per megabase of DNA; similar findings were reported in some uterine corpus endometrial carcinomas and lung adenocarcinomas and squamous cell carcinomas [49]. A high number of mutations are similarly found in melanoma [164]. Evolution within a particular cancer rarely occurs as a process affecting a single, increasingly aggressive clone; instead, the theory of clonal evolution suggests evolutionary processes acting on divergent subclones which evolve simultaneously [165–167]. This divergent evolution can result in substantial intratumoral heterogeneity, which presents a substantial challenge to molecular profiling of tumors. It is a major challenge in cancer research to ensure that minimal samples are taken from clinical specimens while still modelling the inherent heterogeneity with relative accuracy [168].

Epigenetic phenomena also contribute to tumor heterogeneity; however, it is difficult to gain complete understanding of epigenetic heterogeneity and its distinct contributions to cancer progression as the dynamic nature of epigenetic modifications makes them more difficult to profile over time and space. The use of single-cell analytics and our ability to analyze cell-free DNA have begun to unravel some of these complexities and are discussed below. Genomic and epigenomic instability, enabled by the processes of oncogenesis, transformation, progression, and metastasis, alter the phenotypes of individual tumors. While the study of clonal evolution in tumors has been mostly driven by a genetic framework, recent evidence in prostate cancer demonstrates that the epigenome may convergently evolve and contribute to tumor heterogeneity [169]. Also, findings in pancreatic ductal adenocarcinoma have identified that while there is little heterogeneity in driver mutations, epigenetic heterogeneity contributes to metastatic potential [170, 171]. The complex interplay between genetics and epigenetics, which allows some tumors to become invasive and metastatic, supports the use of precision cancer medicine to target driver mutations and epigenetic modifications and the respective changes induced in cellular biology.

Temporal and spatial molecular heterogeneity is a major obstacle to biomarker discovery, and our inability to accurately model and measure heterogeneity presents the greatest obstacle to successful precision medicine in cancer (Figure 2). For example, breast tumors are classified as estrogen receptor- (ER-) positive based on a cut-off of 10% of cells expressing ER; however, a response to therapy may be seen in patients in whom as few as 1% of cells express ER [172]. ER-positivity based on the 10% cut-off does not accurately predict response to selective ER modulators like tamoxifen; thus it is plausible that molecular heterogeneity affects the response of tumors to treatment [173–175]. It is clear that the binary distinction between ER-positive and ER-negative does not differentiate between those who will respond to tamoxifen and those who will not, and molecular heterogeneity may help explain this finding. However, the main challenge in modelling tumor heterogeneity is in obtaining appropriate samples from tumors. Studies have demonstrated that simultaneous evolution takes place among different clones within a tumor, and these may exist within different spaces of the tumor. Specifically, analysis of recurrent gliomas revealed that at least half of the original driver mutations, in classical driver genes such as TP53, were undetectable at recurrence [176]. While biomarker discovery is limited by difficulties in appropriately measuring each clone as it exists within a tumor, techniques such as STAR-FISH that combine the detection of single-nucleotide and copy number alterations at a single-cell level of resolution will support clinical decision-making for precision therapies [177].

Accurately measuring and modelling intratumoral genetic and epigenetic heterogeneity will help determine biomarkers which will indicate if therapy is successful during the course of treatment. One possible approach to determine therapeutic response is by measuring circulating tumor DNA (ctDNA). In one study of melanoma, ctDNA was found to be relatively consistent and informative as a blood-based biomarker [178]. Levels of ctDNA corresponded to response and disease progression. Similarly, a study in breast cancer found that ctDNA predicted metastatic relapse for patients with early-stage disease and was able to predict the genetic events found in the metastatic relapse [179]. Beyond predicting relapse, ctDNA may also offer insight into mechanisms of resistance. For example, RAS pathway mutations have been detected by ctDNA as a mechanism of resistance in colorectal cancer to anti-EGFR therapies [180–182].

Measuring epigenetic alterations is also possible in ctDNA. Detecting methylated SEPT9 ctDNA identified approximately 70% of colorectal cancers [183]. Methylation of ctDNA may be a relatively noninvasive way to measure a patient's response to therapy. For example, the presence of methylated GSTP1 DNA in plasma has been used to track the response of prostate cancer patients [184], and methylation of a panel of ten genes varied between breast cancer patients achieving partial or complete response and those achieving no response to therapy [185]. Many other methylated biomarkers have been established which correlate with disease progression [186–190].

In addition to the growing sensitivity and specificity of measuring cell-free DNA, the advent of single-cell sequencing as a diagnostic tool will improve our understanding of the contributions of genetics and epigenetics to spatial and temporal heterogeneity [191, 192]. Whole-genome bisulfite sequencing [193, 194] or other single-cell analytical techniques including single-cell DNase-sequencing [195] and single-cell chromosome conformation capture (Hi-C) [196, 197] will identify important epigenetic patterns which are relevant to the success of precision therapies. In addition to providing insight into the nature of genetic and epigenetic heterogeneity, these single-cell techniques hold great promise in determining the role of specific subpopulations of tumor cells to cancer initiation and progression. One example of this is the high degree of heterogeneity in expression of the histone linker H1.0 which was demonstrated to support tumor cell self-renewal, an important consideration for the tumorigenicity of tumor cell subpopulations [198].

#### 5.2.1. A Necessary Role for “Omics” Technologies in Molecular Pathology

The current molecular pathology toolbox primarily detects major chromosomal abnormalities including gene amplifications and deletions. Immunohistochemical (IHC) detection of HER2 expression, fluorescent in situ hybridization (FISH) detection of the BCR-ABL translocation, and RT-PCR detection of the PML-RARA translocation have informed clinical use of targeted agents such as trastuzumab, imatinib, and retinoic acid. Our new understanding of cancer as a phenotype influenced by gene expression and modulated by epigenetic factors on top of genetic sequences requires a more detailed view to fully inform the development and selection of targeted therapies. This requires use of more precise tools to determine both the presence and clinical relevance of point mutations and transcriptional modulation (Figure 2). Integrating the use of molecular “omics” technologies is essential to generate a more complete view of the behaviour of cancer and to visualize opportunities for intervention. For example, limited molecular assays such as PCR and Sanger sequencing have been used to detect known cancer mutations, such as V600E in the BRAF gene (Table 1). These higher-resolution diagnostic tools have guided the use of specific BRAF inhibitors including vemurafenib and dabrafenib [199, 200]. The use of “omics” technologies such as complete DNA and RNA sequencing and characterization of protein and metabolite levels are beginning to provide a more comprehensive view of cancer and the unique aberrations in each occurrence (e.g., data emerging from TCGA and ICGC).

Genetics and epigenetics are not two distinct processes; they are intertwined and interregulated at every step. With this in mind, it becomes clear that IHC markers and FISH alone cannot be reliably used for molecular classification of cancer. Molecular pathology that integrates data collected using historic and novel interrogative tools to explore point mutations, epigenetic alterations, gene expression, and posttranslational modifications can provide the information necessary to inform precision therapy (Figure 2).

### 5.3. Resistance to Targeted Therapies

The final challenge to the success of precision medicine is the emergence of resistance to targeted therapies. This is perhaps best modelled by the series of TKIs developed against the* BCR-ABL* translocation [201]. The success of imatinib was dampened by the emergence of resistant variants. As a result, second- and third-generation tyrosine kinase inhibitors were developed against the BCR-ABL gene product [202–205]. Finally, a novel treatment for CML (omacetaxine) was developed, acting independently of the BCR-ABL fusion protein. This has been successful in patients who have failed treatment with earlier BCR-ABL-directed TKIs [206]. We should expect that continuous clonal evolution of cancer cells will enable genetic and phenotypic variants to escape targeted precision therapies.

Primary resistance to precision therapies has been observed with numerous targeted agents. In lung cancer, primary resistance to EGFR- or ALK-targeted tyrosine kinase inhibitors is a result of genetic alterations in or outside of the primary target [207, 208]. The incorporation of genetic and epigenetic information using novel “omics” technologies and computational methods will support the ongoing stratification of patients beyond the absence or presence of a specific alteration (Figure 2). Additionally, in some cases, false-positive results in diagnostics may contribute to observed resistance, as has been documented for ALK rearrangements in lung cancer [209]. Improvements and advancements in diagnostics will eliminate these misinformed selections of therapies. Acquired or secondary resistance broadly results from alterations within the therapeutic target, activation of alternative or downstream signaling, or phenotypic transformation [210]. One approach to combat this is by rational combination of therapeutic agents. For example, combining the small molecule venetoclax with the anti-CD20 rituximab may prevent some acute myeloid leukemias from bypassing BCL2 inhibition [211].

We must attempt to fully map cancer's complex circuitry to stay one step ahead of therapeutic resistance, and this must include ongoing observations of the genetic and epigenetic diversity before, during, and following treatment. The accumulation of data from clinical trials of targeted therapies alone or in combination with existing agents will continue to provide insight into potential approaches to prevent acquired resistance.

## 6. Conclusion

The awareness of precision medicine in the public consciousness has brought out many questions which have yet to be answered. As we target molecular aberrations with increasingly rare occurrences (Figure 1), the cost of drug development forces us to ask how much a cure for cancer, perhaps an individual life, in the foreseeable future, is worth. A precise approach, rather than a personalized approach, to cancer therapy is more cost-effective and is most likely to be realized in any system of socialized medicine. As mentioned previously, the small sample size for specific alterations will limit statistical power and forces the acceptance of new clinical trial designs. An innovative approach to existing medical policy frameworks will strengthen the future for precision medicine by accelerating the pipeline from drug development to approval, and sharing information between major research centers conducting precision medicine trials will similarly hasten the new age of cancer therapy.

On 7 July 2016, US President Barack Obama made a powerful analogy to describe precision medicine, stating “we wouldn't buy a pair of glasses that doesn't match our eyesight, and though plenty of people break their arms, everyone gets fitted for their own cast” [197]. Research focused on exploring the current threats to the success of precision medicine will enable clinical oncology to enter what should be the greatest revolution in medical history, a data-driven precision approach to curing cancer. Our understanding of genetics and epigenetics supports their continued investigation as major contributors to a malignant phenotype and holds the key to unlocking the malignant transformation process so that it can be stopped when it is found.

## Figures and Tables

**Box 1:**
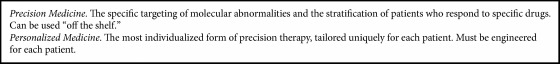
Key terms.

**Table 1:** Selected list of precisely targeted molecular alterations in cancer.

Gene symbol	Gene name	Effect of alteration	Major associations with specific tumor types	Implicated therapy
*Protooncogenes and oncogenes*				
BCR-ABL	Breakpoint cluster region, Abelson murine leukemia viral oncogene homolog fusion protein	Compromises fidelity of DNA repair, deregulates proliferation, impairs apoptosis and differentiation	Chronic myelogenous leukemia	Imatinib, dasatinib,
HRAS/KRAS	Harvey/Kristen rat sarcoma viral oncogene homolog	Constitutively activates MEK/ERK progrowth signaling	Non-small cell lung cancer	Salirasib
BRAF	v-Raf murine sarcoma viral oncogene homolog B	Constitutively activates MEK/ERK progrowth signaling	Melanoma, V600E or V600K mutations	Vemurafenib, dabrafenib
BCL2	B-cell lymphoma/leukemia-2	Impairs apoptosis	Leukemia, lymphoma, melanoma	Venetoclax

*Tumor suppressor genes*				
BRCA1/2	Breast cancer 1/2	Impaired DNA repair	Breast and ovarian cancers	PARP inhibitor (via synthetic lethality)

*Epigenetic modifying genes*				
IDH1/2^*∗∗*^	Isocitrate dehydrogenase	DNA hypermethylation, disrupts differentiation	Acute myeloid leukemia (AML)	AG120, AG221, AG881
EZH2	Enhancer of zeste 2 polycomb repressive complex 2 subunit	Inhibits apoptosis, silences by H3K27 trimethylation	Lymphoma	Tazemetostat
DOT1L	DOT1-like histone H3K79 methyltransferase	Inhibits differentiation and apoptosis	Mixed-lineage leukemia	Pinometostat
DNMT	DNA methyltransferase	Disrupts normal patterns of DNA methylation	Breast and colon cancers, glioma, AML	Azacytidine, decitabine
HDAC	Histone deacetylases	Disrupts normal patterns of histone acetylation	Gastric, breast, colorectal cancers	Vorinostat, romidepsin

*Other targets*				
ER	Estrogen receptor	Sustains proliferative growth signals	Breast and ovarian cancers	Tamoxifen
CD20	B-lymphocyte antigen, cluster of differentiation (CD) 20	Supports B-cell activation and cell cycle progression	B-cell lymphomas	Rituximab
ERBB2 (HER2/neu)	Human epidermal growth factor receptor 2	Sustains proliferative growth signals	Breast, ovarian, uterine, and lung cancers	Trastuzumab
PD1	Programmed cell death protein 1 (CD279)	Prevents activation of T-cells	Potentially targets all solid tumors	Nivolumab
CTLA4	Cytotoxic T-lymphocyte associated protein 4	Prevents activation of T-cells	Potentially targets all solid tumors	Ipilimumab
AP-1	Activating protein 1	Regulates gene expression controlling differentiation, proliferation, and apoptosis	Colorectal cancer *case study*	Irbesartan (angiotensin II receptor antagonist)
PML-RAR	Promyelocytic leukemia, retinoic acid receptor alpha fusion gene	Inhibits granulocytic differentiation	Acute promyelocytic leukemia	Retinoic acid

*Biomarkers for clinical agents*				
APAF1	Apoptotic protease activating factor 1	Prevents apoptosis	Melanoma	Doxorubicin
MGMT	O^6^-methylguanine-DNA methyltransferase	Reverses DNA damage	Glioma	Alkylating agents

^*∗∗*^Although IDH1/2 are not epigenetic modifying genes, alterations in these genes can have profound effects on the epigenome.
